# Risk of Prostate Cancer after Trans Urethral Resection of BPH: A Cohort and Nested Case-Control Study

**DOI:** 10.3390/cancers3044127

**Published:** 2011-11-08

**Authors:** Camilla T. Karlsson, Fredrik Wiklund, Henrik Grönberg, Anders Bergh, Beatrice Melin

**Affiliations:** 1 Department of Radiation Sciences, Oncology, Umeå University, SE-901 87 Umeå, Sweden; E-Mail: beatrice.melin@onkologi.umu.se; 2 Department of Medical Epidemiology and Biostatistics, Karolinska Institute, Stockholm SE-171 77, Sweden; E-Mails: fredrik.wiklund@meb.ki.se (F.W.); henrik.gronberg@meb.ki.se (H.G.); 3 Department of Medical Biosciences, Pathology, Umeå University, SE-901 87 Umeå, Sweden; E-Mail: anders.bergh@medbio.umu.se

**Keywords:** prostate cancer, inflammation, benign prostate hyperplasia, androgen receptor, p53

## Abstract

Epidemiological and experimental evidence suggests that inflammation plays a role in both prostate cancer (PCa) and benign prostate hyperplasia (BPH). This study evaluates the risk of PC after transurethral resection (TURP) for BPH and estimates the PCa risk related to presence of inflammation in the resected material. The Pathology Department at the University Hospital of Umeå (Umeå, Sweden) identified BPH cases (n = 7,901) that underwent TURP between 1982 and 1997. Using these pathological specimens, we compared the incidence of PCa in the cohort to the population and calculated the standardized incidence and mortality ratios (SIR and SMR). Inflammation, the androgen receptor (AR), and p53 were evaluated in a nested case-control study of 201 cases and controls. Inflammation was graded severe or mild-moderate. In the follow-up period after TURP, cases developed prostate cancer and the controls did not. After TURP, SIR for prostate cancer increased [1.26, CI 95% (1.17–1.35) ], whereas SMR decreased [0.59, CI 95% (0.47–0.73) ]. Presence of inflammation at the time of TURP did not differ between cases and controls nor were there differences in p53 or AR staining. The data suggest a small increased risk of PCa after TURP and decreased PCa mortality. Inflammation at the time of TURP is not associated with PCa risk in this material. The increased PCa risk may be attributed to increased surveillance and PSA screening.

## 1. Introduction

Prostate inflammation may be an etiological factor for both benign prostate hyperplasia (BPH) and for prostate cancer [1,2]. Some studies report an increased risk for prostate cancer after TURP [3-5]. On the contrary, several studies [4-8] have reported no increase in the risk of dying from prostate cancer after TURP. BPH and prostate cancer are the most common prostate diseases and share some characteristics: increasing incidence in aging men, dependence on androgens for growth, and response to androgen deprivation therapy. In most cases with BPH, inflammation is present in some part of the prostate and the most common type of inflammation is chronic, often present without symptoms of prostatitis [9]. Acute and chronic prostatitis may develop due to bacteria, viruses, dietary factors, or urine reflux with toxic substances that provoke inflammatory responses [1]. Epidemiological studies focusing on inflammation and the role in cancer development have shown higher prostatitis prevalence in North America compared to Asia [10,11]. Incidence of prostate cancer has a similar geographical distribution and prostatitis could represent an etiological link. In addition, the use of non-steroidal anti-inflammatory drugs seems to lower the risk of developing prostate cancer [12]. Inflammation is uncommon close to and inside prostate cancers [13], but high grade inflammation in tumors is a negative prognostic factor [14] associated with recurrence after radical prostatectomy. Chronic inflammation caused by infections or by other agents are considered to be responsible for 20% of all cancers worldwide [15], and the p53 protein is a key regulator of this process. DNA-damaging stress caused by various agents such as nitric oxide, hydrogen peroxide, or hypoxia related to chronic inflammation may result in mutations [16]. The role of wild-type p53 is to induce apototis among such damaged cells; however, if p53 is mutated, cells with DNA damage will accumulate and tumors may develop. TP53 mutations occur in high frequency in high grade prostate cancers and are less frequent in low grade cancers. Therefore, mutations in TP53 are interpreted as late events in prostate cancer development. So far, the literature is not consistent regarding how often wild type or mutated p53 protein is expressed as measured with immunohistochemistry or how often mutations are detected in BPH.

The androgen receptor (AR) regulates benign prostate and prostate cancer growth. AR expression is affected by inflammation, and in proliferative inflammatory atrophy lesions (PIA) downregulation of AR is common [17]. We investigated the prostate cancer risk togheter with the risk of dying from prostate cancer after TURP performed because of BPH. In addition, we examined whether inflammation and the expression of AR and p53 in the benign prostate at the time of TURP are associated with cancer development.

## 2. Results and Discussion

### 2.1. Cohort Study

In total, the cohort generated 73.900 person-years and there were 700 prostate cancer diagnoses compared to the expected 557.5, which gave the SIR 1.26, CI 95% (1.17–1.35). Median time of follow-up was 9.4 years, maximum 25.9 years. Median-age at TURP was 70.1 years, range 39.9–85 years. Mean time span from TURP to cancer diagnosis was 7.5 years. In the first six months after TURP, the risk of prostate cancer was very high [SIR 4.63, (3.80–5.65) ] and later diminished, but in the long follow-up period, the risk increased again; follow-up for more than ten years displayed a SIR of 1.39, CI 95% (1.22–1.59) (Table 1).

For all ages, the risk of prostate cancer was increased in this cohort, but the risk was more pronounced the younger a patient was at the TURP (Table 2). SIR of prostate cancer in the youngest cohort (age <60 years) was 1.78 CI 95% (1.45–2.19), whereas SIR for the oldest group (>75 years) was more significant, 1.17 CI 95% (0.98–1.40). Mortality rates were significantly decreased; the cohort had 41% lower risk of dying from prostate cancer compared to the population. The decreased mortality was present for all ages, although it was significant only for men 70–74 and 75 or older (Table 2).

### 2.2. Nested Case-Control Study

**Figure 1.**
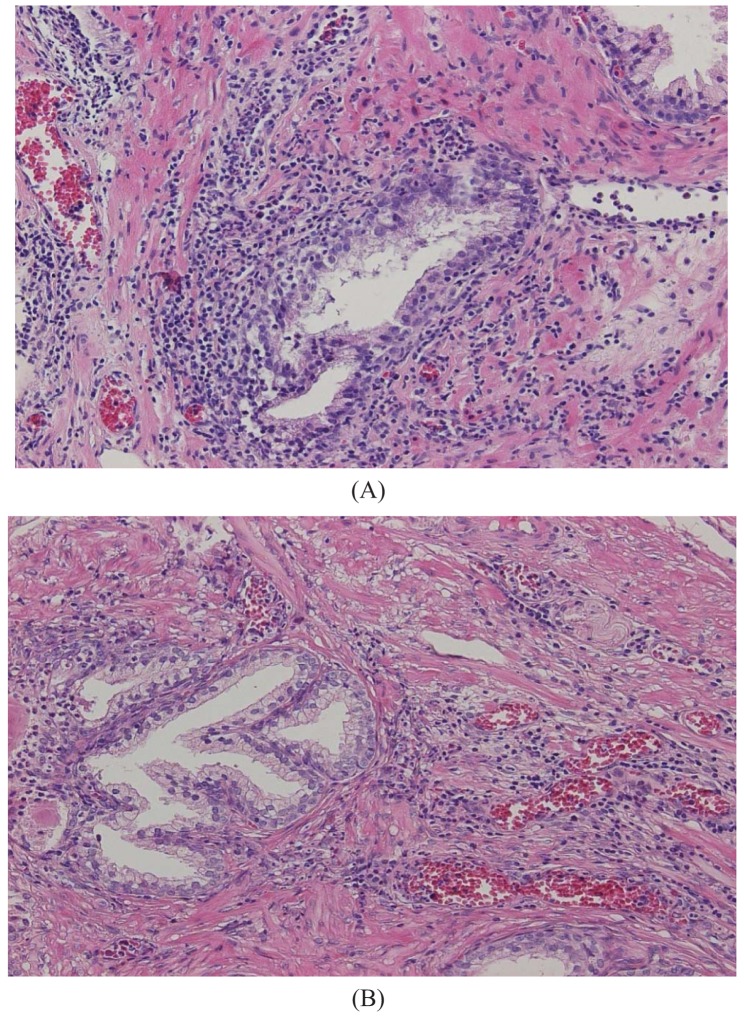
(**A**) Section from a case with severe inflammation adjacent to but not destroying glands (200× magnification); (**B**) Section from a patient with mild to moderate inflammation (200× magnification).

**Figure 2.**
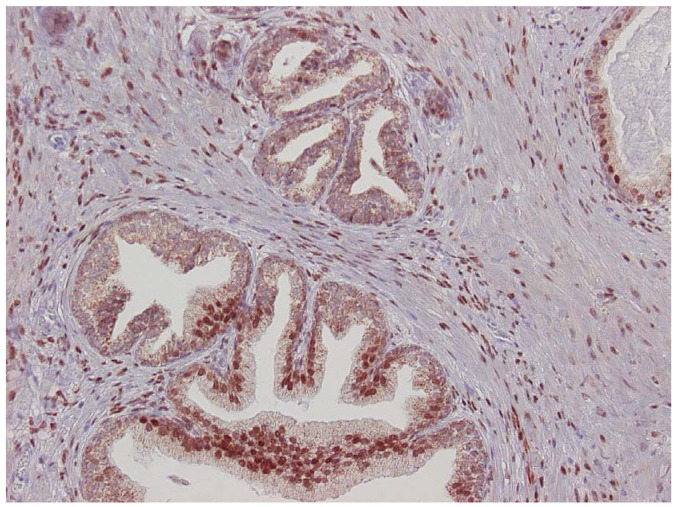
Section from a case showing focal loss of epithelial, but not stroma, androgen receptor expression (brown nuclei, 200× magnification).

The major finding in the case-control study was no association of inflammation to prostate cancer risk, with no significant difference between cases and controls regarding inflammatory extent, and 48.3% of the cases were classified as severe inflammation and the corresponding number for controls was 49.7% (p = 0.83). There were no samples completely devoid of inflammation. Figure 1 shows examples of severe and mild-moderate inflammation. Figure 2 shows a loss of AR expression.

In the small pilot study with different markers for inflammatory cells (CD3, CD20, and CD68), we did not see any difference in patterns or grade between cases and controls nor in cases with severe *vs.* mild inflammation. p53 tended to stain more than expected and there was some loss of AR expression.

In the 50 cases and 50 controls randomly selected from the nested case-control study, the mean percentage of loss or weakened AR expression in cases was 20.2% ± 11.0% SD and in controls, 18.5% ± 14.0% SD. No significant difference between the groups was seen (p = 0.45).

In some specimens, there was loss or low staining of AR in glandular epithelium close to inflammatory patches, but in the nearby stroma, AR was expressed. In specimens with severe inflammation, the AR expression was more affected compared to specimens with mild inflammation, although there was no difference between cases or controls. Positive glandular p53 staining in cases was 14.4% ± 10.0% SD compared to 12.9% ± 11.0% SD in controls (p = 0.52). No difference in staining related to inflammation could be visualized (Table 3).

**Figure 3.**
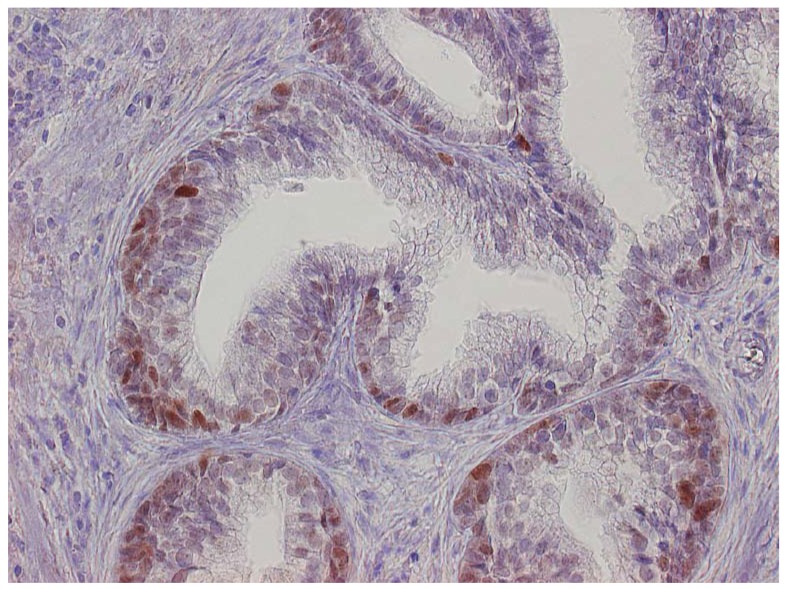
Section form a case showing focal p53 nuclear staining (brown) in the basal epithelial cell layer (400× magnification).

Expression of p53 was exclusively limited to the basal cell layer (Figure 3). The age of the paraffin blocks examined did not affect AR or p53 staining (data not shown).

Of the initial 402 patients selected for the case-control study, 325 (80.8%) underwent one TURP, 62 (15.4%), two TURPs, and remaining 15, three or more treatments. There was no difference between the number of TURPs in cases and controls.

### 2.3. Discussion

In this population-based study of patients undergoing TURP, we found an increased risk of prostate cancer for both early follow-up and after ten years. Very high SIR six months after TURP could probably be attributed to increased surveillance. For example, if the treatment did not reduce symptoms, the patient would be more likely to undergo another operation, increasing the number of cancers detected. Moreover, men with lower urinary tract symptoms (LUTS) are more likely to undergo PC screening and therefore more likely to be diagnosed with prostate cancer. These screenings could actually lead to over-estimating PC in this cohort. In the cohort, 1,772 patients underwent more than one TURP, producing extra tissue available for cancer detection. In the large cohort, patients were excluded from analysis (n = 222) if they already had a prostate cancer diagnosis or were found to have cancer in their first TURP; therefore, LUTS is less likely to be the reason for early excess risk. In fact, removing these men from evaluation possibly creates a selection bias towards lower risk, making the cohort appear healthier compared to the general population where all cancers are included. The 39% excess prostate cancer risk after ten years is intriguing. One possible explanation is that the follow-up period includes the PSA introduction era in Sweden. As a result, patients who have undergone TURP might also be given a PSA test and have more biopsies, resulting in apparant increased risk. Cancers of the prostate are a common feature in the elderly population and many go undetected throughout life, but in the era of PSA testing, more cancers tend to be detected before death [19]. Our findings of excess incidence but lower mortality of prostate cancer following TURP is in line with another large cohort study by Chokkalingam *et al.* [4], who used the Swedish Inpatient Register to collect BPH discharge diagnoses made between 1964 and 1984. Their follow-up period did not cover the 1990s, when the PSA test was introduced, but they found a small but statistically significant increased risk of prostate cancer following TURP. They also found higher SIRs in the 1960s and they comapred the 1970s with the 1980s and hypothesized that removal of larger volumes of prostate tissue over the years due to the development of surgical techniques was responsible for the decreased risk in the later period.

Although our study includes patients in this surgically refined era, we found an increased risk. Moreover, the vast majority of prostate cancers occur in the peripheral zone, an area that TURP minimally resects [20,21]. This characteristic of TURP reduces risk because tissue removal is less probable. Earlier investigations of BPH and prostate cancer risk show divergent results. For example, Armenian *et al.* [3] found a 3.7 increased risk for prostate cancer whereas Greenwald *et al.* [6] did not see any increase. Both these studies were small hospital-based studies with possible sampling bias. In Armenian's study, half of the patients did not have a pathological specimen to exclude prevalent prostate cancer, so many occult cancers may have been missed initially, cancers that later might turn up clinically, inflating the risk. Holman [8] examined the prostate cancer risk and mortality in Australian men following TURP and open prostatectomy. Operated BPH patients had slightly better survival compared with the population and no excess prostate cancer risk. The main goal with that study was to compare outcomes after two surgical procedures, so that study is not fully comparable to our study.

Common findings in these studies [4,6,8] as in ours, with one exception [3], is that TURP-resected patients do not experience increased mortality, which is reassuring for the medical community and patients. Recently, much focus has been on inflammation in the pathogenesis of prostate cancer. Epidemiological data suggest a connection between inflammation and cancer and there is also a link between inflammation and BPH [2]. We did not find evidence for the connection between inflammation and cancer. Inflammation was present in all specimens to a varying extent and grade and that universal presence, together with our two-step grading system for inflammation, may explain the absence of prostate cancer risk association. Our results are similar to a study from the Rotterdam section within the European Randomized Study of Screening for Prostate Cancer (ERSPC) [22]. That study did not find pathological features such as inflammation or PIN in biopsies taken four years preceeding the prostate cancer diagnosis to be predictive. In contrast to these results, MacLennan *et al.* [23] found chronic inflammation to be associated with prostate cancer when they examined biopsies taken because of a suspicion of cancer. They evaluated prediagnostic biopsies for the presence of inflammation and PIN and found cancer more often preceeded with inflammation in the first biopsy. Their findings would agree with the hypothesis of Proliferative Inflammatory Atrophy (PIA) as a precursor of PIN [17], where PIA can be the reaction to inflammation and later turn into PIN or directly into cancer. Unlike our study, these studies used biopsies that sampled the periferal zone rather than the transition zone, the area TURP resects.

Biopsies represent such a small amount of the prostate volume they could lead to sampling bias. Sampling bias can be present in our study as well, as our samples come from the transition zone and cancers arise more often in the peripheral zone. Transition zone cancers have a lower Gleason score, lower proliferation, and less BCL-2 expression compared to peripheral zone cancers and they behave in different ways [24].

In both cases and controls, we found p53 expression to be very common in BPH, a finding that contrasts with other results where p53 is more expressed in high grade cancer [25,26]. Compared to others, we found high p53 expression, a finding that might reflect detection of wild-type p53 showing a well-functioning cell or a very sensitive method. However, the method is not a problem regarding the case-control study as the same evaluation is applied to both groups. The AR is an important regulator for prostate growth and development involved in both BPH and prostate cancer and therefore an obvious candidate to evaluate. Previous investigations have also shown that inflammation causes downregulation of the AR [17,27], a finding confirmed by our study. In spite of the widespread loss or downregulation of the AR, approximately 20% in the present study, no difference could be detected in the nested case-control study. One reason for this could be that the timing of our analysis is too early in the disease development since the mean time to cancer detection was 7.5 years. The AR regulates many processes in prostate cells, including inflammatory response, proliferation, and differentiation [28], and there may be differences between cases and control downstream of the AR. One advantage of our study is the homogeneity of the study group: our selection of patients through the pathological archives meant that all the subjects were operated on and all had a pathological diagnosis. In addition, because our study focused on one region in Sweden, differences in treatment choices regarding BPH were minimal. Until 1991-1992, when medical treatment was introduced in the form of α-blockers and finasteride, few options other than indwelling catheters or surgery were available. Therefore, the vast majority of patients treated for BPH had TURP surgery, which dominated after its introduction in the1970s, and a few had transvesical adenomectomy. For that reason, we think that our cohort is representative of the BPH population. Another strength is the Cancer Registry itself as reporting cancers is mandatory for both clinicians and pathologists, a policy that makes it highly accurate with nearly 100% coverage of Swedish cancer [29]. Limitations are lack of clinical data refering to prostatic diseases such as PSA and Gleason score or comorbidity. Gleason scores among cancer cases would be of interest as they mostly represent low-grade tumors as suggested by the low mortality ratio. PSA became widely used in the beginning of the 1990s in Sweden so that information would not be available for half of the cohort.

## 3. Experimental Section

In a population-based cohort of men with BPH undergoing TURP, we investigated the incidence and mortality of prostate cancer. Using a nested case-control study, we also explored whether inflammation, AR, and p53 expression were associated with development of prostate cancer.

The Department of Pathology at the University Hospital of Umeå (northern Sweden) serves approximately 500,000 inhabitants. All archival specimens from patients who had undergone a trans-urethral resection (TURP) of the prostate between 1982 and 1997 (n = 10,129) were included in the study. All patients were diagnosed as having BPH. In 1,772 cases, more than one TURP was performed on an individual patient, but only the first TURP was used in the analyses. Patients were excluded if there was a previous or concomitant diagnosis of prostate cancer (n = 222). Excluded were men older than 85 at time of TURP (n = 234). After these exclusions, the cohort size was 7,901 men. The cohort was followed from the date of TURP until death, age of 85, or 2 December 2008, whichever came first, and person years were calculated using the program PYRS (IARC, Lyon, France). Incidence and mortality of prostate cancer in the whole cohort was compared to the population in the northern region of Sweden. Standardized Incidence Ratio (SIR) and Standardized Mortality Ratio (SMR) were defined as the ratio between the observed and the expected number of cases. The expected number of prostate cancer in the cohort was calculated by multiplying the calendar and age-specific prostate cancer incidence rate for northern Sweden by person-years. Incidence rates for northern Sweden from 1982 through 2007 were obtained from the Cancer Registry of Northern Sweden, part of the Swedish Cancer Registry. SMR was calculated in a similar procedure using the causes of death registry for northern Sweden. The difference in SMR calculations from SIR calculations was that duration of follow-up lasted until 31 December 1998. The shorter follow-up time was due to centralisation of the Causes of Death Registry after that time. Two-tailed 95% confidence interval (CI) of the SIR and SMR was calculated using Byar's formula.

From the initial cohort, a nested case-control study was conducted to evaluate the impact of inflammation at the time of TURP on the risk of developing prostate cancer. The cases selected were ≤75 years and had at least a six-month time span between the first TURP and cancer diagnosis. This selection was carried out to avoid the first clinical work-up time after TURP where more cancers might be detected and allow for sufficient follow-up time. Controls were matched by age, calendar year of TURP, and follow-up time. Controls were defined to be alive at the time when their corresponding case got prostate cancer. The inclusion criteria resulted in 270 cases and 201 matched controls; therefore, all 201 cases with a matching control were selected. Of 402 selected cases and controls, 379 specimens were available in the archives. Re-examining the pathological specimens revealed 25 misclassifications of the type of specimen. There were 20 cases and five controls who had biopsies instead of TURP. For 18 of the TURP specimens, a cancer was missed in the first clinical evaluation. As cancer in the initial TURP session was an exclusion criterion, these specimens and the biopsies were withdrawn from further analysis, leaving 336 specimens for evaluation (149 cases and 187 controls).

To classify inflammation in the BPH specimens, we used a dichotomous scale. The definition of inflammation was mild-moderate or severe inflammation. Definition of severe inflammation was infiltration of inflammatory cells covering one-third or more of the specimens with either diffuse or multifocal pattern of inflammatory cells. Inflammatory cells should be present in confluent sheets in at least three large different tissue chips or covering one-third of the slide. Patterns of inflammatory tissue destruction, defined as a disruption of the epithelium, fell into the severe category. The mild to moderate inflammation was defined as areas of confluent sheets of cells or very small ones, no tissue destruction, and less than a third of the area covered. Our definition of severe corresponds to the consensus classification by Nickel *et al.* [18] of moderate and severe grade together with multifocal and diffuse extent. Our classification of mild to moderate relates to all other patterns defined by Nickel.

In this group of cases, a small pilot study of ten cases with controls were randomly selected and evaluated for different inflammatory cell markers—CD3, CD 20, and CD68, corresponding to T-cells, B-cells, and macrophages—and for AR and p53. Cases and controls were compared regarding patterns and distribution of the different cells. For each of the inflammatory cell types, we evaluated distribution, extent, and grade according to the proposed consensus by Nickel *et al.* [18]. After the pilot study, we randomly selected 50 cases and their corresponding controls. From this group five cases and two controls were missing in the archives, making it 45 cases and 48 controls. This was done to investigate whether differences in immunohistochemistry patterns of the Androgen Receptor (AR) or p53 could be a risk factor for developing prostate cancer. Normally, both luminal epithelial and stromal cells express AR in the nucleus. To evaluate loss or weakened expression of AR, we counted 30–40 luminal epithelial cells in 10–15 visual fields throughout the specimen at 400× magnification. Definition of loss was no staining in the nucleus; weakened expression was defined as less than half of the staining intensity compared to adjacent normal staining glands.

For evaluation of p53, we first counted 100 glands in every specimen and glands with a p53 positive cell were considered positive, creating a percentage To grade the extent and strength of p53 staining, we created an index by multiplying number of glands with p53 positive cells with percentage of positive cells in each gland and staining strength of p53. Staining strength was for weak (1) or strong (2) staining in each gland. We used the Fisher's exact test to evaluate differences between cases and controls regarding inflammation. Differences in expression of AR and p53 were assessed using the non-parametric Mann-Whitney test.

### 3.1. Immunohistochemistry

Immunohistochemistry (IHC) for each antigen was performed with the Ventana BenchMark automated staining system. All paraffin embedded specimens were sectioned into 5 μm sections and put onto slides, deparaffinised, and rehydrated. Antigen retrieval of AR, CD3, CD68, and CD20 was done by heating the sections in a microwave oven in EDTA (pH8) buffer; for p53, antigen retrieval was done by heating sections in a citrate buffer (pH 6) at 900 W for 20 min. Primary antibodies were revealed with the Ventana iVIEW DAB detection kit. The following antibodies and their concentrations were used: p53, Oncogene (Ab-6) 1:200 San Diego CA, USA; AR, BioCare 1:50 Concord, CA USA; CD3, NovoCastra, UK (NCL-CD3-565) 1:100; CD20, Ventana ready-to-use Tucson, AZ, USA; and CD68 Dako (MO814) 1:2000 Stockholm, Sweden.

## 4. Conclusions

In conclusion, we have found an increased risk of prostate cancer following TURP, but not increased mortality, a disparity that might be explained by follow-up in the PSA era, by diagnosis of clinically non-significant cancers, or by unidentified factors. Inflammation, androgen reseptor, or p53 expression in resected tissue does not seem to be associated with prostate cancer risk in this material.

## Figures and Tables

**Table 1.** SIR and SMR for prostate cancer after TURP for BPH related to duration of follow-up.

**Follow-up months**	**Obs.**	**Exp.**	**SIR 95% CI**	**Obs.**	**Exp.**	**SMR 95% CI**
<6	98	21.2	4.63 (3.80–5.65)	1	8	0.13 (0.02–0.91)
6–11	14	21.2	0.66 (0.39–1.12)	2	8	0.26 (0.06–1.02)
12–59	145	169.9	0.85 (0.73–1.00)	25	59	0.42 (0.29–0.63)
60–119	222	186.3	1.19 (1.04–1.36)	39	47	0.83 (0.60–1.13)
120+	221	158.9	1.39 (1.22–1.59)	14	16	0.87 (0.51–1.47)
All	700	557.5	1.26 (1.17–1.35)	81	138.0	0.59 (0.47–0.73)

TURP: Trans Urethral Resection of the Prostate; BPH: Benign Prostate Hyperplasia; CI: Confidence Interval; SIR: Standardized Incidence Ratio; SMR: Standardized Mortality Ratio.

**Table 2.** SIR and SMR for prostate cancer after TURP for BPH by age at TURP.

**Age at TURP**	**Obs.**	**Exp.**	**SIR 95% CI**	**Obs.**	**Exp.**	**SMR 95% CI**
<60	91	51.0	1.78 (1.45–2.19)	3	3.4	0.89 (0.29–2.77)
60–64	143	101.3	1.41 (1.20–1.66)	9	12.4	0.73 (0.38–1.40)
65–69	184	156.9	1.17 (1.02–1.36)	26	30.6	0.85 (0.58–1.25)
70–74	161	145.2	1.11 (0.95–1.29)	25	43.5	0.57 (0.39–0.85)
75+	121	103.0	1.17 (0.98–1.40)	18	48.1	0.37 (0.24–0.59)
All ages	700	557.5	1.26 (1.17–1.35)	81	138.0	0.59 (0.47–0.73)

CI: Confidence Interval; SIR: Standardized Incidence Ratio.

**Table 3.** Case and control: expression of AR and p53 in TURP specimens.

	**Case**	**SD%**	**Control**	**SD%**	**p-value**
	**n= 45**		**n = 48**		
**AR**	20.2%	11.0	18.5%	14.0	0.45
**p53**	14.4%	10.0	12.9%	11.0	0.52

AR: Androgen receptor; SD: standard deviation; For this group randomly selected from the initial 402, five cases and two controls were missing in the archives, therefore the total number is not 50/50.
